# Three-dimensional analysis of dental arch forms in Italian population

**DOI:** 10.1186/s40510-018-0233-1

**Published:** 2018-09-10

**Authors:** Bruno Oliva, Simone Sferra, Anna Lucia Greco, Francesco Valente, Cristina Grippaudo

**Affiliations:** 1 Institute of Catholic University of Sacred Heart, Largo Francesco Vito 1, 00198 Rome, Italy; 2 University G. d’Annunzio of Chieti-Pescara, Via dei Vestini 31, 66100 Chieti, Italy

**Keywords:** Arch form, Dental arch depth, 3D analysis, 3D software

## Abstract

**Background:**

The aim of this study was the comparison of male and female upper and lower dental arch form in untreated Italian patients by 3D analysis, to find differences in shape, in transversal and longitudinal diameters between sexes, and to give a representative set of population’s dental arch to clinicians in order to provide suitable orthodontic treatment.

**Methods:**

The sample consisted of 3D scans of dental casts deriving from 104 Italian untreated patients (Male = 35, Female = 69) in permanent dentition. An evaluation of the arch form was performed by angular and linear values on every patient using a 3D software (SolidWorks®). A Student’s two-tailed *t* test was used to determine if the differences in measurements between the male and female groups were significant and the level of significance was set at *P* < 0.05.

**Results:**

Statistically significant differences in upper and lower transversal and longitudinal diameters were found. Male arch widths were significantly larger than those of females. Male intercanine, intermolar, and interpremolar diameters were significantly greater than females. Dental arch depth was significantly smaller in the female group. Anterior upper dental arch form was flatter, wider, and less sharp in the female group.

**Conclusions:**

Basing on the anatomical arches differences found between sexes concerning Italian patients, it is suggested to have regard to each patient pre-treatment arch form, width, and depth during orthodontic treatment according to gender.

**Electronic supplementary material:**

The online version of this article (10.1186/s40510-018-0233-1) contains supplementary material, which is available to authorized users.

## Background

Preformed archwires mimic the form of the dental arch, and are the most preferred wires by orthodontists worldwide. Currently, orthodontic archwires are manufactured in several different dental arch forms, as it allows the practitioner to choose the most suitable wire for each patient [1–3].

Despite numerous investigations [4, 5], there remains contradictory evidence regarding the ideal size and shape of an orthodontic arch form. For years, researchers have been trying to define the “ideal” arch form. It is a common assumption that the dental arch is symmetric in nature and can be represented by an algebraic or geometric formula [6–8]. Additionally, it has long been suggested that considerable variability occurs in the arch forms of different types of malocclusions [9–12], which may preclude the effective use of a single ideal arch form for all cases. Camporesi [13], studying the arch form in Italian population, proved that none of the analyzed commercial archwires fit perfectly with the arch form of the studied patients, that is with the exception of the Brader arch form. Nevertheless, with the continued development of computer-assisted analysis [14, 15], the approach of custom designing arch forms may provide the optimum solution for accurately describing the ideal orthodontic arch form for the individual patient.

Relative to gender, it has been documented that males and females exhibit different skeletal facial dimensions [16–18], differences in multiple facial characteristics [19], as well as differences in maxillary and mandibular arch width [20].

To the knowledge of the authors, as for the Italian population is concerned, few studies have evaluated dental arch form [8, 13, 21, 22]. Thus, limited literature of analysis on the Italian population exists, especially regarding the distinction between the shape and dimension of dental arches between males and females; additionally, arch length and width have not been considered combined indicators of arch dimensions. Notably, there has yet to exists a unique method of analysis presented within the various studies.

In a previous study, Grippaudo et al. [23] used a method of analysis that took into consideration the evaluation of the dental arch form with dental landmarks positioned on occlusal digital photos of plaster dental casts. The limitation of the afore study is the impossibility to carry out linear measurements through digital photos without a controlled 1:1 proportion between the subject pictured and the actual subject. On the other hand, angular measurements and linear ratio remain unaltered. In the present study, to overcome these limitations, the authors bore an improvement of the analysis using a computer-assisted analysis: it allowed to visualize three-dimensional images of dental casts and to effect linear and angular measurements in order to obtain standardize and replicable data.

The aim of this study was to compare male and female upper and lower dental arch form in untreated Italian patients, through 3D analysis. The expected result was to find out differences in shape, in transversal and longitudinal dimensions between sexes. The observations might lead to give a representative set of Italian population’s dental arch according to patient’s gender, since dental arch length and width have both been considered unlike previous studies.

The null hypothesis stated that between males and females, there were no differences in the maxillary and mandibular dental arch form and dimensions.

## Methods

### Sample

One hundred four dental casts from patients of both genders (males and females), seeking orthodontic treatment at the Dental Clinic of the Catholic University of the Sacred Heart (Rome, Italy), were randomly selected for a 3D scan; both maxillary and mandibular arches. The Ethic Committee approved all the aspects and steps of this research (No#1811).

The studied sample had the following inclusion criteria: both dental arches; class I malocclusion; age range between 23 and 48 years old; state of permanent dentition of both dental arches with all teeth (except third molars) fully erupted to the occlusal plane without edentulous spaces; and dental displacement up to 3 mm (corresponding to a state of perfect alignment and minimal irregularity) in accordance with Little’s Irregularity Index [24]. The exclusion criteria were history of medical complications or syndromes, history of craniofacial malformation, dental trauma, oral breathing habits, orthodontic treatment, or maxillofacial surgery.

The sample was divided into two groups according to sex: Male = 35, Female = 69. The sample size was calculated to be *N* > 21 based on a study confidence level of 95%, study power level of 90%, standard deviation of 2 mm or degrees, and difference in mean values within 2 mm or degrees.

The following equation [25] was used for sample size calculation:

$$ N>\frac{2{\left({Z}_{\alpha }+{Z}_{1-\beta}\right)}^2{\sigma}^2}{\Delta ^2} $$

Where *N* is the calculated sample size for group, *Z*_*α*_ is a constant and is the percentage point of the normal distribution corresponding to the two sided significance level (if significance level is 5%, *Z*_*α*_= 1.96), *Z*_1 − *β*_ is a constant set by convention according to power of the study (if the power is 90%, *Z*_1 − *β*_ = 1.28), σ are standard deviations (estimated), and *∆*^2^is the difference between the mean values (estimated effect size).

Dental measurements taken on digital models can be more replicable, and significantly faster, than those done manually on traditional plaster models [14, 26, 27]. Hence, for the present study, 3D scans (.STL extension) were obtained through the digitalization (Dental Scanner, scanSystems, Pisa, Italy) of gypsum dental casts, which were developed from polyvinylsiloxane impressions of both dental arches for each patient, then subsequently analyzed. The accuracy of the digital cast was compared with that of the stone cast by measuring the distance between four anatomic landmarks. Differences were assessed using intraclass correlation coefficient (ICC). Dental Scanner by ScanSystem is highly reliable with ICC ranging from 0.926 to 0.999.

### Landmarks definition

**Fig. 1:**
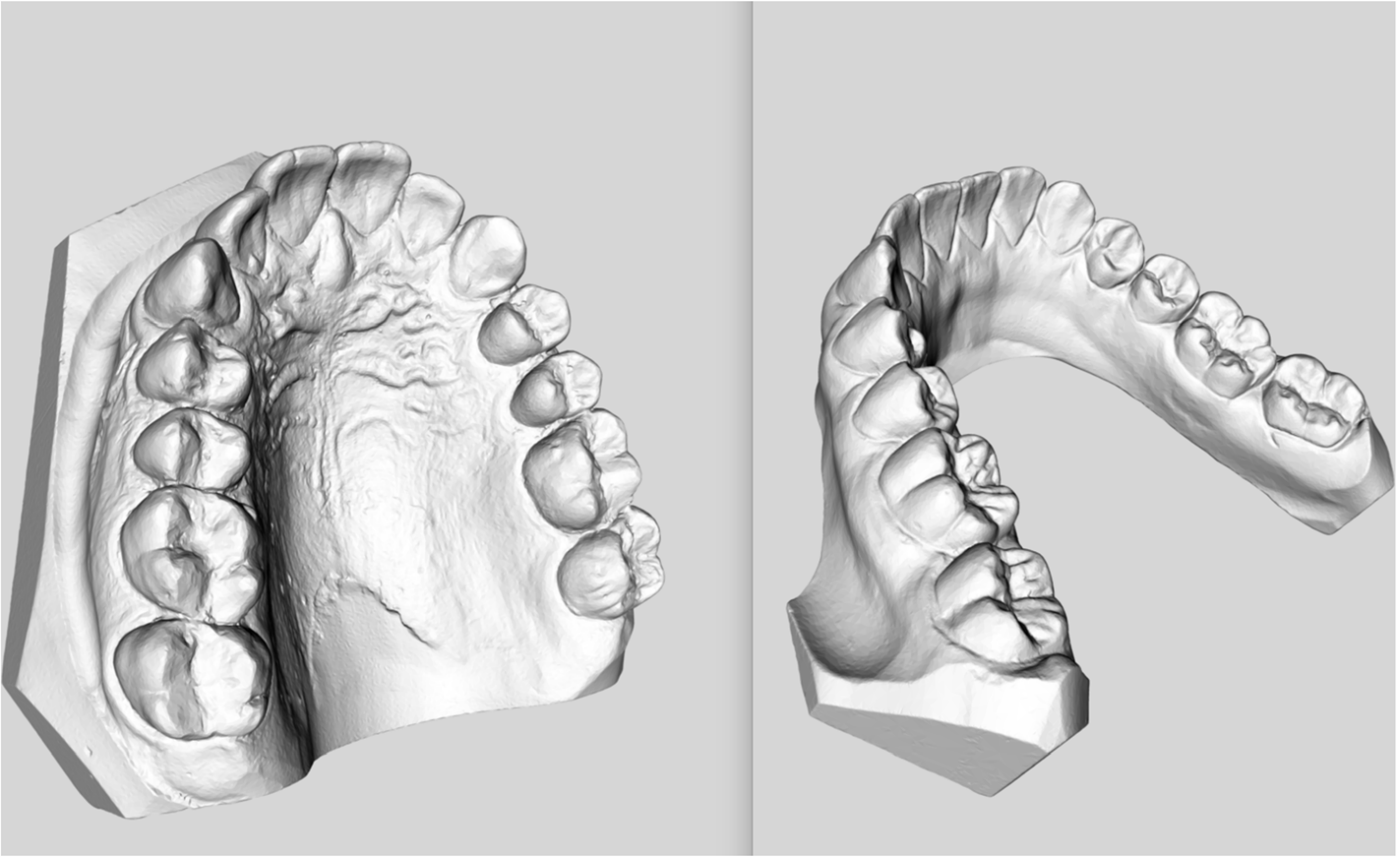
Illustrative example of the 3D images of jaws models by SolidWorks®

3D scans were analyzed by a specialized 3D planning software (SolidWorks**®** 2013 for Windows, Dassault Systemes SolidWorks Corp., Waltham, Massachusetts, USA) (Fig. 1), which allowed for an evaluation of the dental arch forms and dimensions. The software permits different movement of the digital dental casts, so it is possible to view the precise allocation of landmarks. Due to the integration of different points of view, a detailed evaluation can be conducted.

For the purpose of this study, linear and angular measurements were used for the general assessment. A reference plane parallel to the occlusal plane was set on the 3D scan model, both in the maxilla and in the mandible. The model was then oriented in an occlusal point of view. From this viewpoint, a pentagon (lying on the reference plane) inscribed inside the arches was drawn. A vertex of the pentagon was then placed between the two central incisors, two other vertices lied on the cusp of the canines, and the other two were later placed at the center of the occlusal face of the first molars.

### Variables

**Fig. 2:**
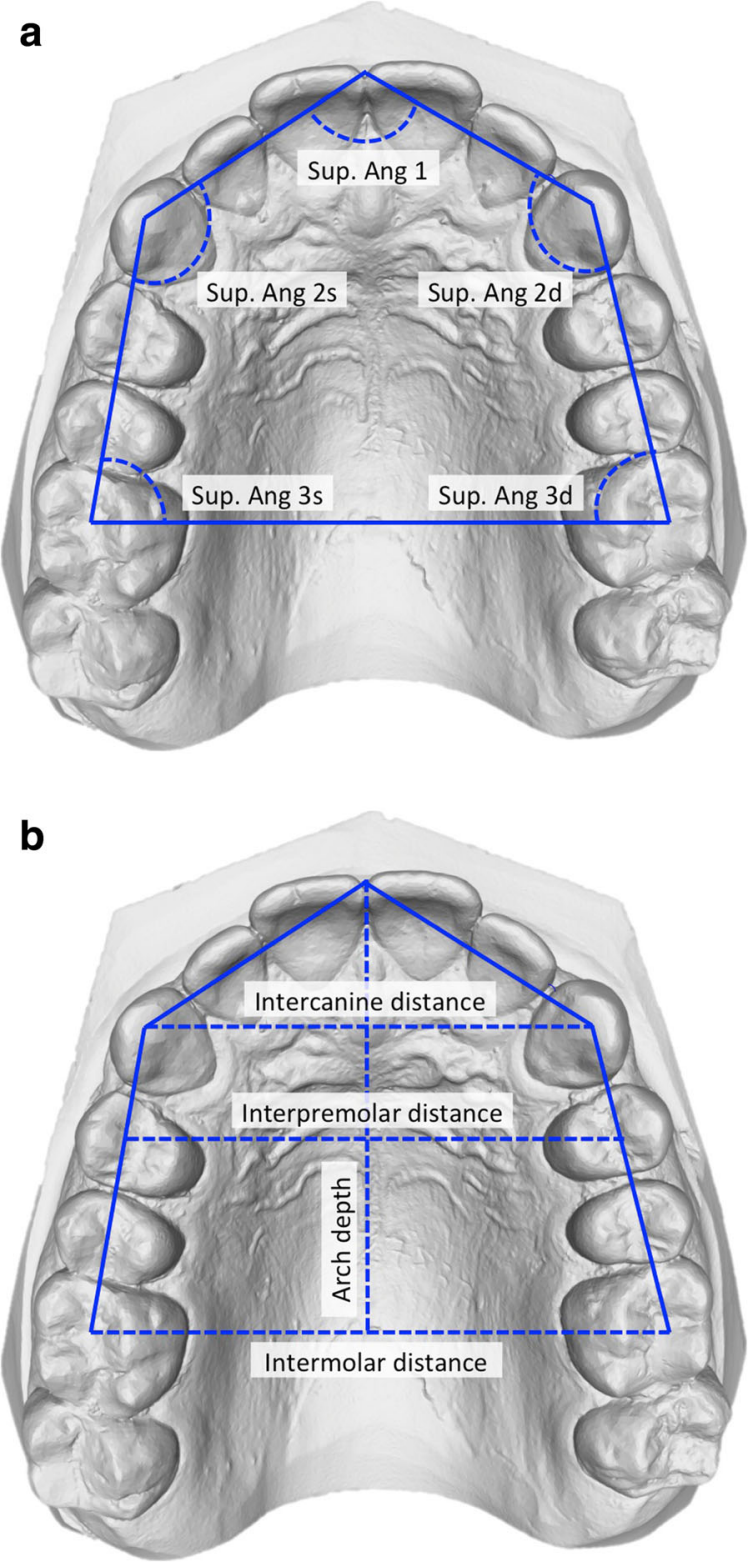
**a**, **b** Method and variables for the shape analysis of the maxilla

**Fig. 3:**
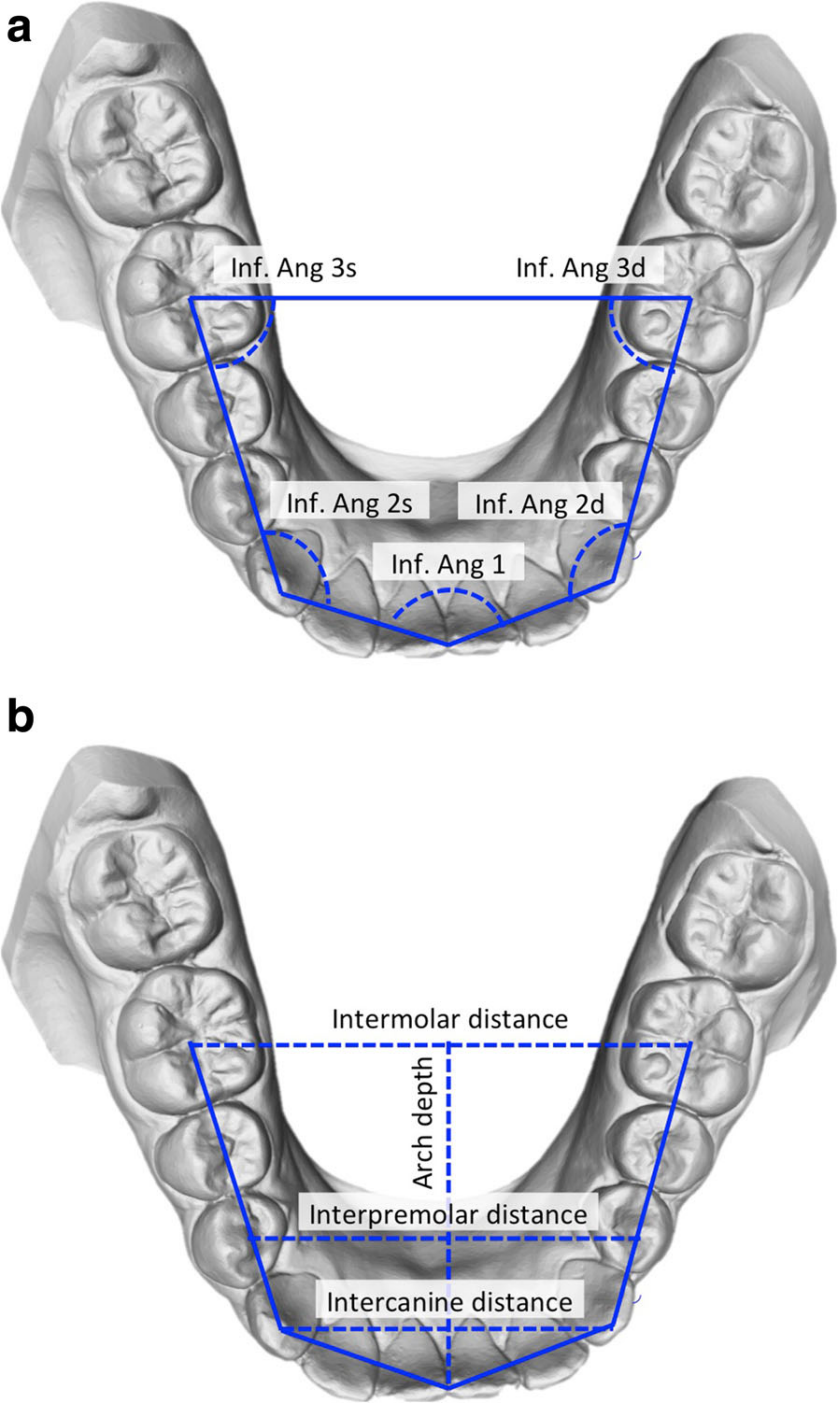
**a**, **b** Method and variables for the shape analysis of the mandible

For the present study, the variables of analysis were (Figs. 2a, b and 3a, b):

- Five internal angles of the pentagon (*Superior Angle 1*, *2d*, *2s*, *3d*, *3s in the maxilla*; *Inferior Angle 1*, *2d*, *2s*, *3d*, *3s in the mandible*);
- *Intercanine distance* (the distance between the two canines cusps);
- *Interpremolar distance* (the distance between the two first premolars vestibular cusps);
- *Intermolar distance* (the distance between the occlusal face center of the two first molars);
- *Intercanine*/*intermolar distance ratio;*
- *Arch depth* (the distance from the anterior vertex of the pentagon to the line connecting the occlusal face center of the two first molars).

One calibrated operator who was blinded to the subjects’ age and sex drew the pentagon and assessed the variables. All the tracings were checked for accuracy by a second investigator. The analysis of the variables was performed in an independent manner on both upper and lower dental arches.

### Statistical analysis

The assessment of the data distribution type was performed by the Shapiro-Wilk test. The results revealed a normal distribution of the data. Descriptive statistics were calculated for all of the considered variables. The two-tailed Student *t* test was applied to find the differences between the two groups and a *P* value of 0.05 was considered as the threshold to detect statistically significant differences. All of the statistical analysis was performed using a specific statistical software (MatLab**®** for Windows, MathWorks Inc., Natick, Massachusetts, USA).

### Method error

Twenty 3D scans were randomly selected and measured again by the same operator 1 week after the initial measurement. To calculate the measurement error, the Dahlberg’s formula [28] was applied. The Dahlberg error was 0.01 mm for linear measures and 0.02° for angular measurements.

## Results

Table 1 reports descriptive statistics (mean, standard deviation, median, minimum and maximum) for each variable evaluated in the maxilla and in the mandible. All measurements except the ratio between the intercanine and intermolar distance are reported as angular values (°) and linear values (mm).

**Table 1:** Descriptive analysis

	Number	Minimum	Maximum	Median	Mean	Standard deviation (SD)
Maxilla						
*Sup*. *ang*. *1*^a^	104	105.2	155.5	125.6	126.4	9.4
*Sup*. *ang*. *2d*^a^	104	110.1	149.7	131.8	131.3	6.5
*Sup*. *ang*. *2s*^a^	104	115.1	145.9	132.1	132.4	6.5
*Sup*. *ang*. *3d*^a^	104	67.7	89.0	74.9	75.1	4.0
*Sup*. *ang*. *3s*^a^	104	66.1	83.3	74.6	74.7	3.4
Interpremolar distance^b^	104	29.2	45.8	36.8	37.2	2.4
Intercanine distance^b^	104	29.0	37.9	33.1	33.2	2.2
Intermolar distance^b^	104	32.7	51.7	45.4	45.3	2.9
Intercanine-intermolar distance ratio	104	0.6	1.1	0.7	0.7	0.1
Arch depth^b^	104	25.2	36.7	31.7	31.4	2.5
Mandible						
*Inf*. *ang*. *1*^a^	104	107.6	172.4	137.2	138.0	12.4
*Inf*. *ang*. *2d*^a^	104	113.4	157.8	129.0	130.6	9.6
*Inf*. *ang*. *2s*^a^	104	103.9	152.4	130.8	130.5	8.1
*Inf*. *ang*. *3d*^a^	104	60.9	84.4	70.8	70.7	4.2
*Inf*. *ang*. *3s*^a^	104	59.5	78.4	69.5	69.9	3.3
Interpremolar distance^b^	104	24.2	34.3	29.8	29.9	2.1
Intercanine distance^b^	104	20.6	30.6	25.7	25.5	1.8
Intermolar distance^b^	104	34.2	46.4	41.1	41.2	2.5
Intercanine-intermolar distance ratio	104	0.5	0.8	0.6	0.6	0.0
Arch depth^b^	104	20.6	32.5	26.9	27.0	2.3

^a^Degrees (°); ^b^Millimeters (mm)

Table 2 reports the mean and standard deviation of the same variables divided in the two groups: males and females. All measurements except the ratio between the intercanine and intermolar distance are reported as angular values (°) and linear values (mm). It showed the level of significance related to each variable calculated by two-tailed Student’s *t* test.

**Table 2:** Mandible and maxilla *T* test between male and female

	Male (*n* = 35)		Female (*n* = 69)		Significance (*P*)
	Mean	SD	Mean	SD	(*T* test)
Maxilla					
*Sup*. *ang*. *1*^a^	123.76	7.81	127.65	9.85	0.031*
*Sup*. *ang*. *2d*^a^	133.14	4.40	130.42	7.17	0.099
*Sup*. *ang*. *2s*^a^	133.93	6.06	131.59	6.55	0.118
*Sup*. *ang*. *3d*^a^	74.62	3.15	75.58	4.33	0.239
*Sup*. *ang*. *3s*^a^	74.59	2.89	74.94	3.72	0.679
Interpremolar distance^b^	38.70	2.01	36.39	2.18	0.000*
Intercanine distance^b^	34.43	1.87	32.66	2.08	0.000*
Intermolar distance^b^	47.41	2.76	44.28	2.51	0.000*
Intercanine-intermolar distance ratio	0.73	0.03	0.74	0.07	0.389
Arch depth^b^	32.76	2.05	30.86	2.51	0.000*
Mandible					
*Inf*. *ang*. *1*^a^	137.04	12.02	137.91	12.51	0.626
*Inf*. *ang*. *2d*^a^	131.45	9.43	130.47	9.63	0.479
*Inf*. *ang*. *2s*^a^	130.68	8.24	130.46	8.16	0.854
*Inf*. *ang*. *3d*^a^	69.94	2.65	70.80	4.17	0.146
*Inf*. *ang*. *3s*^a^	70.18	3.41	69.90	3.30	0.561
Interpremolar distance^b^	30.93	1.76	29.91	2.07	0.000*
Intercanine distance^b^	26.13	1.59	25.45	1.84	0.010*
Intermolar distance^b^	42.59	2.39	41.17	2.48	0.000*
Intercanine-intermolar distance ratio	0.61	0.04	0.62	0.04	0.439
Arch depth^b^	27.81	2.66	27.00	2.33	0.014*

^a^Degrees (°); ^b^Millimeters (mm); ^*^significant

In the maxilla, the differences between the angular measurements were not statistically significant, with the exception of the *Sup*. *Ang*. *1* (*P* ≤ 0.05). This angular value resulted greater in the female group. All the linear measurements showed statistically significant differences between the two groups (*P* < 0.05), and these measurements were greater in the male group than in the female one.

In the mandible, none of the angular measurements were significant. On the contrary, every difference between linear measurements was significant (*P* ≤ 0.05). As in the maxilla, these measurements were greater in males than in females.

In both the maxilla and the mandible, the intercanine/intermolar distance ratio was not significant.

Therefore, it was clearly demonstrated that males have significantly greater maxillary and mandibular dental arch widths and depth than females (*P* ≤ 0.05). Dental arch forms remain similar between the two groups.

## Discussion

Findings from our investigation demonstrated that there is a significant difference in male and female arch dimensions; however, it cannot be stated that there is a statistically significant difference in arch form between the two sexes, with the exception of the anterior maxillary region. Therefore, the study hypothesis was partially rejected.

According to the literature, the form of the adult male and female face is significantly different from both a qualitative and quantitative point of view: the difference involves both hard and soft tissue components [16–19, 29, 30]. A sexual dimorphism in dental arch form was therefore expected in this study, with the male teeth occupying the jaw in a different form.

The study’s results showed a statistically significant gender difference for arch variables with linear measurement; however, this was not the case for variables with angular measurement. This included both arches. The exceptions were (i) the *intercanine*/*intermolar distance ratio* and (ii) *Sup*. *Ang*. *1*, which showed significant differences between the sexes. It therefore showed that males, on average, had larger transversal and longitudinal dimension of both arches than females.

With regard to the transverse dimension, these results were in agreement with those of a previous study by Forster and coworkers [31]. In the study mentioned, however, arch depth was not analyzed: this variable was considered and measured in the present study, being indicative of the arch longitudinal dimension. Comparatively analyzing the outcome of the variable *intercanine*/*intermolar distance ratio*, the similarities between the two studies are remarkable. The authors derived from Forster’s study data the *intercanine*/*intermolar distance ratio* for both males and females. Foster found values of 0.73 in the maxilla and 0.61 in the mandible. In the present study, the ratio was detected to be in the maxilla 0.73 for both sexes and in the mandible 0.61 for males and 0.62 for females. The *intercanine*/*intermolar distance ratio* did not significantly differ between sexes; it can therefore be confirmed that the arch shape between males and females is strongly similar.

It can be noticed that in both studies, the arch transversal dimensions are significantly greater in males than females; however, the *intercanine*/*intermolar ratio* was not significantly greater in males than in females. Additionally, in the present study, the arch depth is significantly greater in males than females: it is therefore possible to point out how arch form keeps an average stability when there are changes in arch dimensions.

Another important finding from the results was a statistically significant difference *Sup*. *Ang*. *1* between sexes. In females, this value on average was greater than males. This can be interpreted as a sign of different morphology of the anterior maxillary arch area between sexes, taking into account the strong similarity of all other angular variables and the *intercanine*/*intermolar distance ratio*. Therefore, the maxilla appeared to be more ovoid and flatter in females than in males. This result led to the hypothesis that the dentition of females tends to be less protruded and more upright in bone bases than males. This is in agreement with findings by Christie [20]. Maxillary arch depth in females was minor, when comparing to males, with 30.81 ± 2.49 mm in females and 32.76 ± 2.05 mm in males, for the different measure of the *Sup*. *Ang*. *1*. When this angle of the pentagon becomes more acute, on average parity of the other corners, the arch depth inevitably increases; in contrast, when said angle becomes more obtuse, the arch depth decreases. Males therefore have on average a maxillary arch than females deeper of 1.95 mm, with a *Sup*. *Ang*. *1* on average 4° more closed.

Other researchers in agreement with the dental arch size difference between sexes are Eroz et al. [32], Moyers et al. [33], and the aforementioned Christie [20]. Moyers et al. [33] and Christie [20], as in the case of both Forster [31] and the present study, analyzed subjects in which the growth phase was over, differently from Eroz et al. [32]. In the study by Eroz et al [32], among the different parameters analyzed, the values of the *intermolar distance* was found to be sex related: in males, this value was on average greater. Christie [20] showed that the *intermolar distance* and the *arch depth* were greater in males than females, and that there were gender-related skeletal dimensional differences: the whole jaw, posterior facial height, cranial front height, was greater in males than in females. The results of the present study confirmed that female arches are smaller than male arches [17, 34, 35]. Therefore, in accordance with Bhowmik et al. [3], the outcomes of this study suggest not using the same orthodontic wires for male and female patients, as doing so would give greater expansion in female patients, leading to greater post-treatment instability.

In regards to the arch shape, this study’s results were in concordance with those found by Ferrario et al. [8] and Camporesi et al. [13], which concluded that in Caucasians, considering the arch form, regardless of size, there were no differences in the form of both arches between the sexes. It is interesting to observe how the conclusions of these two works are similar notwithstanding the different methods of analysis used: Ferrario et al. [8] applied the matrix of the Euclidean distance, whereas Camporesi et al. [13] applied the thin-plate spline analysis (TPS).

The presented investigation showed that the difference between male and female dental arches is just a size difference and not a whole shape difference.

Patients were selected with permanent dentition because relatively rapid changes occur during the transitional dentition. Once a functional permanent dentition is established, smaller changes continue to be observed [36–41].

In the presented method of analysis, the points (vertexes of the pentagon) were placed on references even if there was a light dental crowding or displacement. For future studies, it could be useful to identify the role of dental crowding related to arch form.

Therefore, it would also be interesting for the authors to analyze the independent behavior of the different variables influencing arch form between sexes: considering malocclusion and vertical facial patterns, clustering age range, and dental crowding.

## Conclusions

This study was conducted to examine the difference in shape of male and female upper and lower dental arches, as well as the difference in their transversal and longitudinal diameters through a 3D analysis, in untreated Italian patients.

Within the limitations of the study, the conclusions can be summarized as follows:

- Italian males on average have a greater transversal and longitudinal dimension of both arches than females;
- There are no statistically significant differences in dental arch form between sexes, with the exception of the anterior maxillary area;
- Due to the anatomical arch differences found between sexes, it is suggested to have regard to each patient pre-treatment dental arch form, width, and depth during orthodontic treatment in accordance with gender.

## Additional file

Additional file 1: Data analysed during this study. (XLSX 35 kb).

## References

[1] Al-Barakati RG, Alqahtani ND, AlMadi A, Albarakati SF, ALKofide EA (2016). Evaluation of the fit of preformed nickel titanium arch wires on normal occlusion dental arches. Saudi Dent J.

[2] Regragui S, Boulif H, Rerhrhaye W (2016). Study of the adaptability of preformed orthodontic archwires to the average dental arch form of a Moroccan population. Int Orthod.

[3] Bhowmik SG, Hazare PV, Bhowmik H (2012). Correlation of the arch forms of male and female subjects with those of preformed rectangular nickel-titanium archwires. Am J Orthod Dentofac Orthop.

[4] Lombardo L, Fattori L, Molinari C, Mirabella D, Siciliani G (2013). Dental and alveolar arch forms in a Caucasian population compared with commercially available archwires. Int Orthod.

[5] Lombardo L, Coppola P, Siciliani G (2015). Comparison of dental and alveolar arch forms between different ethnic groups. Int Orthod.

[6] MacConaill MA, Scher EA (1949). The ideal form of the human dental arcade, with some prosthetic application. Dent Rec (London).

[7] Currier JH (1969). A computerized geometric analysis of human dental arch form. Am J Orthod.

[8] Ferrario VF, Sforza C, Miani A, Tartaglia G (1993). Human dental arch shape evaluated by Euclidean-distance matrix analysis. Am J Phys Anthropol.

[9] Uysal T, Memili B, Usumez S, Sari Z (2005). Dental and alveolar arch widths in normal occlusion, class II division 1 and class II division 2. Angle Orthod..

[10] Slaja M, Spaljb S, Pavlinc D, Illesd D, Slaje M (2010). Dental archforms in dentoalveolar class I, II and III. Angle Orthod.

[11] Isik F, Nalbantgil D, Sayinsu K, Arun T (2006). A comparative study of cephalometric and arch width characteristics of class II division 1 and division 2 malocclusions. Eur J Orthod.

[12] Lombardo L, Setti S, Molinari C, Siciliani G (2013). Intra-arch widths: a meta-analysis. Int Orthod.

[13] Camporesi M, Franchi L, Baccetti T, Antonini A (2006). Thin-plate spline analysis of arch form in a southern European population with an ideal natural occlusion. Eur J Orthod.

[14] Grünheid T, Patel N, De Felippe NL, Wey A, Gaillard PR, Larson BE (2014). Accuracy, reproducibility, and time efficiency of dental measurements using different technologies. Am J Orthod Dentofac Orthop.

[15] Huanca Ghislanzoni L, Lione R, Cozza P, Franchi L (2017). Measuring 3D shape in orthodontics through geometric morphometrics. Prog Orthod.

[16] Ingerslev CH, Solow B (1975). Sex differences in craniofacial morphology. Acta Odontol Scand.

[17] Chung CH, Wong WW (2002). Craniofacial growth in untreated skeletal class II subjects: a longitudinal study. Am J Orthod Dentofac Orthop.

[18] Wei SH (1970). Craniofacial width dimensions. Angle Orthod..

[19] Tanikawa C, Zere E, Takada K (2016). Sexual dimorphism in the facial morphology of adult humans: a three-dimensional analysis. HOMO- J Comp Hum Biol.

[20] Christie TE (1977). Cephalometric patterns of adults with normal occlusion. Angle Orthodontist.

[21] Ferrario VF, Sforza C, Colombo A, Carvajal R, Duncan V, Palomino H (1999). Dental arch size in healthy human permanent dentitions: ethnic differences as assessed by discriminant analysis. Int J Adult Orthodon Orthognath Surg.

[22] Defraia E, Baroni G, Marinelli A (2006). Dental arch dimensions in the mixed dentition: a study of Italian children born in the 1950s and the 1990s. Angle Orthod..

[23] Grippaudo C, Oliva B, Greco A, Sferra S, Deli R (2013). Relationship between vertical facial patterns and dental arch form in class II malocclusion. Prog Orthod.

[24] Little RM (1975). The irregularity index: a quantitative score of mandibular anterior alignment. Am J Orthod.

[25] Bhalerao S, Kadam P (2010). Sample size calculation. Int J Ayurveda Res.

[26] Garino F, Garino GB (2002). Comparison of dental arch measurements between stone and digital casts. World J Orthod.

[27] Gracco A, Buranello M, Cozzani M, Siciliani G (2007). Digital and plaster models: a comparison of measurements and times. Prog Orthod.

[28] Dahlberg G (1940). Statistical methods for medical and biological students. Br Med J.

[29] Halazonetis DJ, Shapiro E, Gheewalla RK, Ernest Clark R (1991). Quantitative description of the shape of the mandible. Am J Orthod Dentofac Orthop.

[30] Scheideman GB, Bell WH, Legan HL, Finn RA, Reisch JS (1980). Cephalometric analysis of dentofacial normals. Am J Orthod.

[31] Forster CM, Sunga E, Chung CH (2008). Relationship between dental arch width and vertical facial morphology in untreated adults. Eur J Orthod.

[32] Eroz UB, Ceylan I, Aydemir S (2000). An investigation of mandibular morphology in subjects with different vertical facial growth patterns. Aust Orthod J.

[33] Moyers RE, van der Linden FPGM, Riolo ML, McNamara JA Jr. Standards of human occlusal development. Monograph 5, Craniofacial Growth Series. Ann Arbor: Center for Human Growth and Development, The University of Michigan; 1976.

[34] Merz ML, Isaacson RJ, Germane N, Rubenstein LK, Diego S (1991). Tooth diameters and arch perimeters in a black and white population. Am J Orthod Dentofacial Orthop.

[35] Sinclair PM, Little RM (1985). Dentofacial maturation of untreated normals. Am J Orthod.

[36] Carter GA, McNamara JA (1998). Longitudinal dental arch changes in adults. Am J Orthod Dentofac Orthop.

[37] Bishara SE, Jakobsen JR, Treder JE, Stasl MJ (1989). Changes in the maxillary and mandibular tooth size-arch length relationship from early adolescence to early adulthood. A longitudinal study. Am J Orthod Dentofac Orthop.

[38] Bishara SE, Treder JE, Jakobsen JR (1994). Facial and dental changes in adulthood. Am J Orthod Dentofac Orthop.

[39] Sillman JH (1964). Dimensional changes of the dental arches: longitudinal study from birth to 25 years. Am J Orthod.

[40] Lee RT (1999). Arch width and form: a review. Am J Orthod Dentofac Orthop.

[41] Harris EF (1997). A longitudinal study of arch size and form in untreated adults. Am J Orthod Dentofac Orthop.

